# Engineered Bacterial Outer Membrane Vesicles Hitchhiking on Neutrophils for Antibody Drug Delivery to Enhance Postoperative Immune Checkpoint Therapy

**DOI:** 10.1002/advs.202505000

**Published:** 2025-04-27

**Authors:** Meng Guan, Xiao‐Ting Xie, Dong Zhou, Kai Cheng, Bin Zhang, Xin‐Yue Xu, Yong Li, Yi‐Tong Zhou, Wei Peng, Li‐Li Chen, Peng‐Shuo Dong, Si Chen, Jia‐Hua Zou, Bo Liu, Yuan‐Di Zhao, Jin‐Xuan Fan

**Affiliations:** ^1^ Britton Chance Center for Biomedical Photonics at Wuhan National Laboratory for Optoelectronics – Hubei Bioinformatics & Molecular Imaging Key Laboratory Department of Biomedical Engineering College of Life Science and Technology Huazhong University of Science and Technology Wuhan Hubei 430074 P. R. China; ^2^ Department of Oncology Huanggang Central Hospital of Yangtze University Huanggang Hubei 438000 P. R. China; ^3^ Hubei Clinical Medical Research Center of Esophageal and Gastric Malignancy Huanggang Hubei 438021 P.R. China; ^4^ Hubei Key Laboratory of Plasma Chemistry and Advanced Materials School of Material Science and Engineering Wuhan Institute of Technology Wuhan Hubei 430205 P. R. China; ^5^ Key Laboratory of Biomedical Photonics (HUST) Ministry of Education Huazhong University of Science and Technology Wuhan Hubei 430074 P. R. China

**Keywords:** engineered bacteria, immunotherapy, neutrophils, outer membrane vesicles

## Abstract

In clinical practice, surgical removal of tumors often leaves behind small tumors and circulating tumor cells, increasing the risk of metastasis and recurrence, which seriously affects treatment outcomes. Immunotherapy activates the immune system to monitor and inhibit tumor metastasis and recurrence long‐term. However, inflammatory microenvironments at surgical sites lead to immunosuppressive tumor‐associated macrophages (TAMs), causing immune evasion. Additionally, tumor cells overexpress the immune checkpoint CD47, further weakening the phagocytic and cytotoxic functions of macrophages. Here, the bacterial outer membrane vesicles (OMV) hitchhiking on neutrophils are utilized to precisely deliver immune checkpoint blockade antibodies to the tumor resection site. Escherichia coli is reprogrammed to express CD47 antibody and used to extract CD47 antibody‐containing OMV, followed by insertion of Ce6 photosensitizer into the membrane (OC47‐Ce6). Purified autologous neutrophils phagocytose and carry OC47‐Ce6 for precise targeting to the postoperative tumor resection site, mediating tumor cell killing, aCD47 release, and tumor‐associated antigen presentation by light. In vitro and in vivo experiments demonstrate that OC47‐Ce6 enhances TAM phagocytic function through TAM polarization and CD47 blockade. This approach effectively activates T‐cell anti‐tumor immune responses and significantly reduces the risk of postoperative tumor recurrence and metastasis.

## Graphical abstract

This study employs neutrophils for the precise delivery of anti‐CD47 antibodies, combined with bacterial outer membrane vesicles, to enhance macrophage phagocytic function in the tumor microenvironment. This approach effectively activates T‐cell anti‐tumor immune responses and significantly reduces the risk of postoperative tumor recurrence and metastasis, providing an effective strategy for tumor immunotherapy.

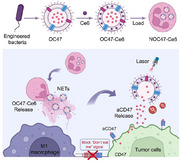

## 1 Introduction

Surgical excision is one of the crucial therapeutic approaches in tumor treatment. Despite the continuous advancements in surgical techniques, postoperative metastasis and recurrence remain significant challenges.^[^ ^1^ ^]^ Therefore, there is an urgent need to explore more effective therapeutic strategies. Immunotherapy activates the body's immune system to sustain long‐term suppression of tumor cells and holds promise as a key therapeutic approach for preventing postoperative cancer recurrence and metastasis.^[^ ^2^ ^]^ However, the inflammatory response and wound healing at the surgical site can lead to immune suppression, thereby limiting the effectiveness of immunotherapy.^[^ ^3^ ^]^ Although surgery can remove the tumor and its associated tumor‐associated macrophages (TAMs), the postoperative inflammatory environment attracts a large number of immune‐suppressive macrophages.^[^ ^4^ ^]^ These cells secrete anti‐inflammatory factors such as IL‐10, thereby creating an immune‐suppressive microenvironment that promotes the escape of residual tumor cells, accelerating their metastasis and recurrence. To address this issue, reprogramming TAMs from an immunosuppressive phenotype to M1 macrophages with antitumor activity represents an effective strategy. Additionally, CD47 binds to the signal regulatory protein α (SIRPα) on the surface of macrophages, forming a “don't eat me” signal that helps tumor cells with high CD47 expression evade phagocytosis by immune cells.^[^ ^5^ ^]^ Therefore, combining TAMs reprogramming with immune checkpoint blockade can more effectively enhance the phagocytic capacity of TAMs, breaking their immunosuppressive state.

As innate immune cells, macrophages can be sensitized by immune adjuvants, such as Toll‐like receptor (TLR) agonists, to activate immune cytotoxic effects.^[^ ^6^ ^]^ However, such activation must be spatiotemporally controlled to avoid the side effects of systemic immune activation. As a naturally derived nanomaterial, bacterial outer membrane vesicles (OMV) have been widely used as carriers for specific local immunomodulation within the tumor microenvironment (TME).^[^ ^7^ ^]^ OMVs retain bacterial antigenicity, which can be recognized by the immune system to elicit host immune responses, and they carry pathogen‐associated molecular patterns (PAMPs) from donor bacteria to activate various TLR signaling pathways, while significantly reducing safety concerns.^[^ ^8^ ^]^ In addition, engineered bacteria can secrete OMVs containing relevant proteins, thereby enhancing the functionality of OMVs.^[^ ^9^ ^]^ As a result, OMV are considered highly promising tumor vaccine delivery vectors.^[^ ^10^ ^]^ However, OMV lack targeting capability and requires modifications or transporters to assist in targeting the tumor site for immunomodulation.^[^ ^11^ ^]^ We observed that the postoperative microenvironment, in addition to recruiting macrophages, also attracted a significant number of neutrophils (NEs) due to its highly inflammatory nature. Therefore, utilizing NEs as multifunctional OMV carriers can achieve precise and controlled local immunomodulation at the tumor site post‐surgery while minimizing the risk of systemic immune responses.^[^ ^12^ ^]^

**Figure 1:**
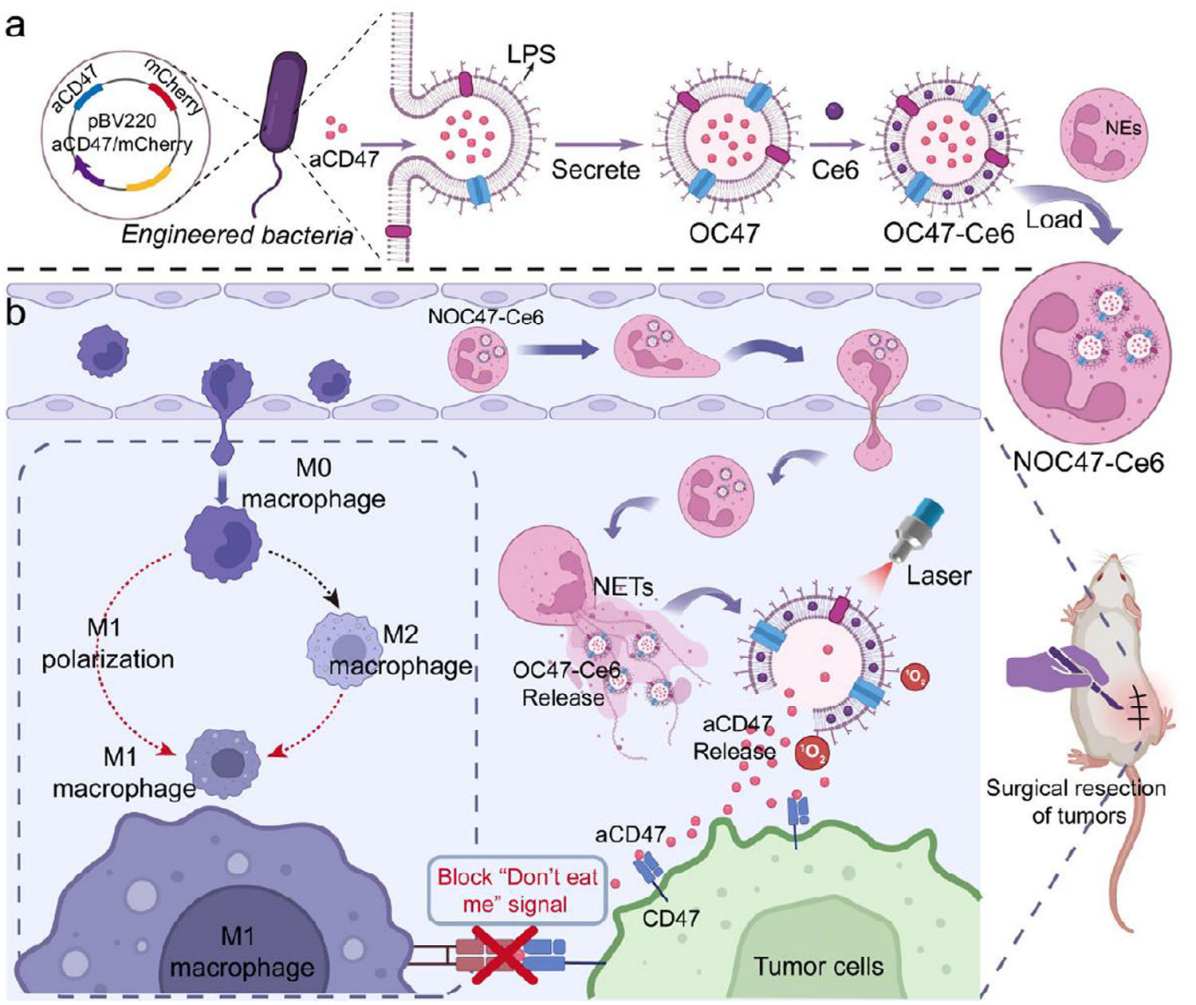
Synthesis and antitumor mechanism of NOC47‐Ce6. a) Schematic diagram of the synthesis of NOC47‐Ce6. b) Schematic diagram of engineered bacterial OMV hitchhiking on NEs to deliver antibody drugs, enhancing the antitumor mechanisms of postoperative immune checkpoint therapy. Figure 1 was created with BioRender.com released under a Creative Commons Attribution 4.0 International license (https://creativecommons.org/licenses/by/4.0/).

Here, we report a targeted strategy employing engineered bacterial OMV hitchhiking on NEs to activate the phagocytic function of TAMs via TAM reprogramming and immune checkpoint blockade, aimed at inhibiting postoperative recurrence and metastasis of breast cancer (**Figure** **1**). We obtained OMV containing the CD47 antibody (OC47) from cultured, genetically modified Escherichia coli and loaded the small molecule drug Ce6 onto OC47 (OC47‐Ce6). NEs then internalized OC47‐Ce6 to create a NE‐based delivery vector (NC47‐Ce6). Following surgical removal of the tumor, the site undergoes an inflammatory response characterized by the release of inflammatory factors. We administered NC47‐Ce6 via tail vein injection, leveraging its migration along the inflammation gradient to the tumor site. Once NC47‐Ce6 reaches the tumor site, NEs, stimulated by excessive inflammatory factors, release neutrophil extracellular traps (NETs), thereby releasing OC47‐Ce6. OC47‐Ce6 can simultaneously bind to CD47 on tumor cells and TLRs on TAMs, promoting TAM polarization to the M1 state and blocking the “don't eat me” signal on tumor cells, thereby stimulating TAMs to phagocytose tumor cells effectively. Concurrently, under laser irradiation, Ce6 generates reactive oxygen species (ROS), further killing tumor cells and causing the release of numerous tumor‐associated antigens. These antigens are processed and presented by antigen‐presenting cells to draining lymph nodes, subsequently triggering a T cell‐mediated antitumor immune response, effectively preventing postoperative tumor recurrence and metastasis.

## 2 Results

### 2.1 Construction and Characterization of NOC47‐Ce6

**Figure 2:**
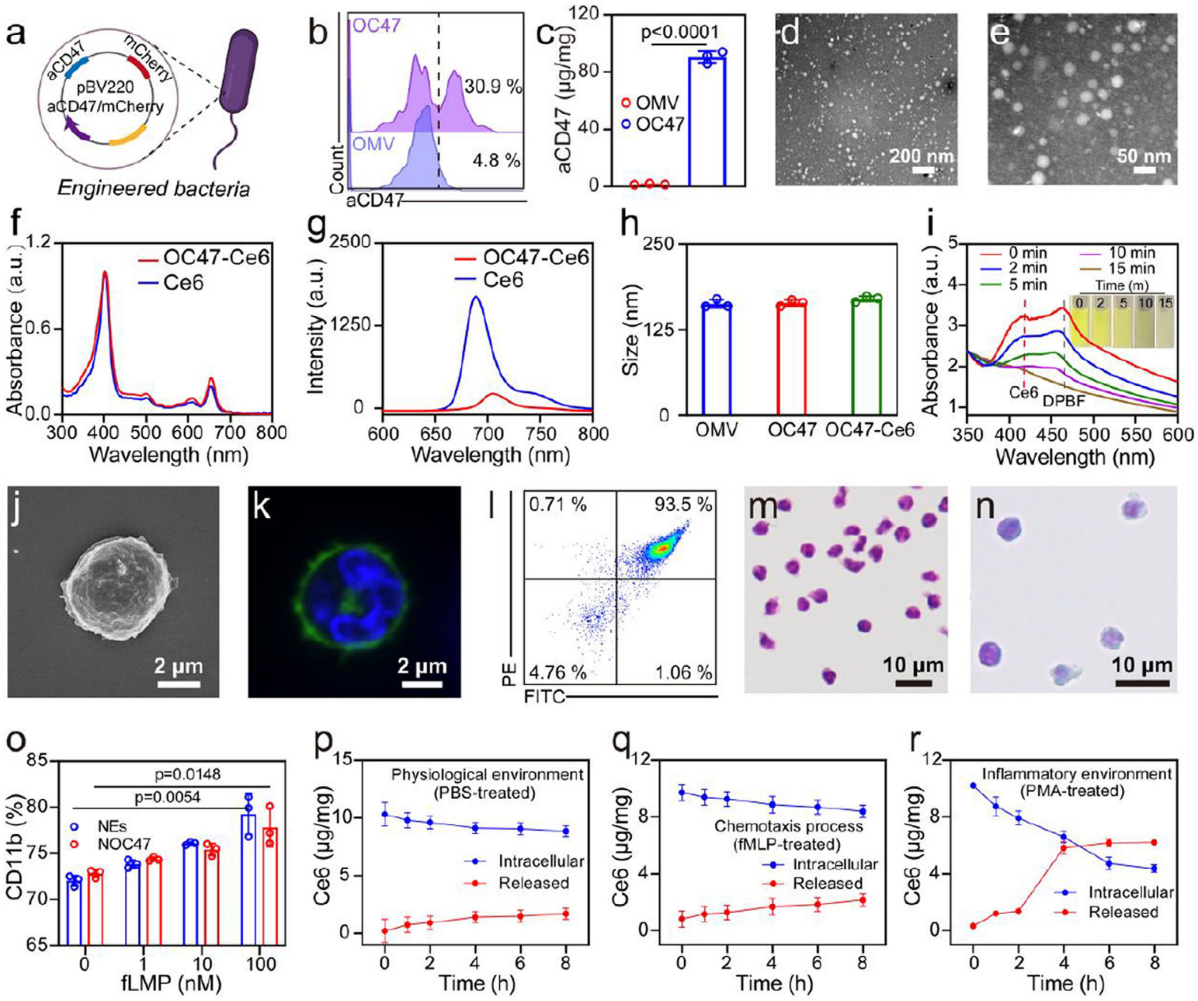
Construction and Characterization of NOC47‐Ce6. a) Schematic diagram of M‐aCD47 expression plasmid. b) Flow cytometry was used to detect the fluorescence intensity of aCD47 in bacteria. c) Content of aCD47 in each milligram of OMV and OC47. Data presented as mean ± SD, n = 3. d, e) TEM images of OC47. Scale bar: 200 nm (d) and 50 nm (e). f) UV–vis absorption spectra of OC47‐Ce6 and Ce6. g) Fluorescence spectra of OC47‐Ce6 and Ce6. h) Hydration diameter of OMV, OC47 and OC47‐Ce6. Data presented as mean ± SD, n = 3. i) Decomposition of DPBF in a solution containing DPBF and OC47‐Ce6 after different durations of 606 nm laser irradiation. j) SEM images of NEs. Scale bar: 2 µm. k) Confocal staining of NEs nuclear membrane dyes. Scale bar: 2 µm. l) Flow cytometry analysis of the purity of NE double stained with FITC conjugated Gr‐1 and PE‐conjugated MAIR‐IV antibodies. m, n) Giemsa staining of NEs. Scale bar: 10 µm (m) and 10 µm (n). o) Changes in CD11b expression levels on NEs and NOC47 cell membrane after treatment with different concentrations of fMLP for 1 h. Data presented as mean ± SD, n = 3. p–r) Measuring the amount of Ce6 released and retained from NOC47‐Ce6 over time in the presence of PBS (p), fMLP (10 nM) (q), and PMA (100 nM) (r). Data presented as mean ± SD, n = 3. The significance between the two groups in (c) was assessed by unpaired two‐tailed Student's t‐test. Figure 2a was created with BioRender.com released under a Creative Commons Attribution 4.0 International license (https://creativecommons.org/licenses/by/4.0/).

To generate OC47, we inserted the DNA fragment encoding the CD47 antibody into the expression plasmid pBV220, transfected it into Escherichia coli MG1655, and named the resultant strain M‐aCD47 (**Figure** **2**a). SDS‐PAGE was used to determine the total protein content in the original E. coli (MG1655) and OMV (derived from the original MG1655). Outer membrane protein A (OmpA), an important component of OMV, exhibited characteristic OmpA bands on the SDS‐PAGE gel, indicating that OMV retained the major bacterial protein components (Figure S1, Supporting Information). Flow cytometry confirmed the expression of the CD47 antibody in OC47 (derived from the M‐aCD47) (Figure 2b). Additionally, the precise content of aCD47 in OC47 was determined to be 90 µg mg^−1^ using an ELISA kit (Figure 2c). Transmission electron microscopy (TEM) revealed that OC47 obtained via ultracentrifugation had a uniform morphology with a size range of ≈30–50 nm (Figure 2d,e). The Ce6 solution was incubated with OMV at 4 °C in the dark for 2 h, allowing Ce6 to stabilize within the phospholipid bilayer of the OMV through hydrophobic interactions. Subsequently, multiple rounds of centrifugation were performed using a 100 kDa molecular weight cut‐off filter to remove unbound Ce6, resulting in a final concentration of 100 µg of Ce6 per 1 mg of OMV. The successful loading of Ce6 into OC47 was confirmed by UV–vis (Figure 2f) and fluorescence spectroscopy (Figure 2g). Dynamic light scattering (DLS) analysis indicated that there was no significant change in the hydrodynamic size of OMV, OC47, and OC47‐Ce6 (Figure 2h). DPBF (1,3‐diphenylisobenzofuran) reacted with singlet oxygen to produce a colorless product, resulting in a decrease in absorbance. With increasing illumination time, the absorbance of the DPBF and OC47 mixed solution at 410 nm gradually decreased (Figure 2i). However, in the absence of OC47‐Ce6, the light bleaching of DPBF at the same concentration under light exposure was relatively mild (Figure S2, Supporting Information). This indicated that Ce6 generates ROS upon illumination, leading to the bleaching of DPBF and thereby demonstrating the photodynamic activity of OC47.

NEs were isolated and purified from mouse peripheral blood using density gradient centrifugation. Scanning electron microscopy (SEM) clearly showed that NEs were round or oval in shape, with surface microvilli and pseudopodia, which facilitated migration and phagocytosis of pathogens (Figure 2j). Under confocal microscopy, NEs exhibited typical lobulated nuclei, with microvilli and pseudopodia visible at the cell edges (Figure 2k). Flow cytometry analysis showed a purity of 93.5% (Figure 2l). NEs were incubated with OC47, and the resulting NOC47 was collected by centrifugation, achieving a loading capacity of 25 µg OC47 per 10^7^ cells. After loading OC47, no significant morphological changes were observed in the NEs (Figure 2m,n). The ability of NOC47 to respond to inflammation was evaluated, including specific protein expression and chemotaxis. CD11b is a surface protein specific to NEs that regulates adhesion and migration, and its expression is upregulated during inflammation. Following treatment with various concentrations of N‐formylmethionyl‐leucyl‐phenylalanine (fMLP), a significant increase in CD11b expression on NOC47 was observed at 100 nM fMLP (Figure 2o; Figure S3, Supporting Information). No significant difference in CD11b levels was observed between NOC47 and NEs. Furthermore, Transwell migration assays demonstrated that NOC47 exhibited fMLP‐activated chemotaxis comparable to that of NEs. This migratory ability of NOC47 was dependent on the concentration of fMLP (Figure S4, Supporting Information). In summary, NOC47 retained the physiological activity of NEs, and NOC47 could actively respond to inflammatory stimuli and migrate directionally to inflammation sites. Next, we investigated the stability and release of OC47‐Ce6 within NEs under different conditions. PBS, fMLP, and phorbol myristate acetate (PMA) were used to simulate normal physiological conditions, chemotaxis, and inflammatory sites, respectively. After treatment with PBS (Figure 2p) and fMLP (Figure 2q), very little OC47‐Ce6 was released from NEs. In contrast, NOC47‐Ce6 showed a burst release of OC47‐Ce6 after incubation with PMA for 4 h (Figure 2r). This indicated that NOC47‐Ce6 could achieve effective drug release at inflammation sites, enabling precise therapy.

### 2.2 OC47 Enhances M1 Polarization and Tumor Cell Phagocytosis

**Figure 3:**
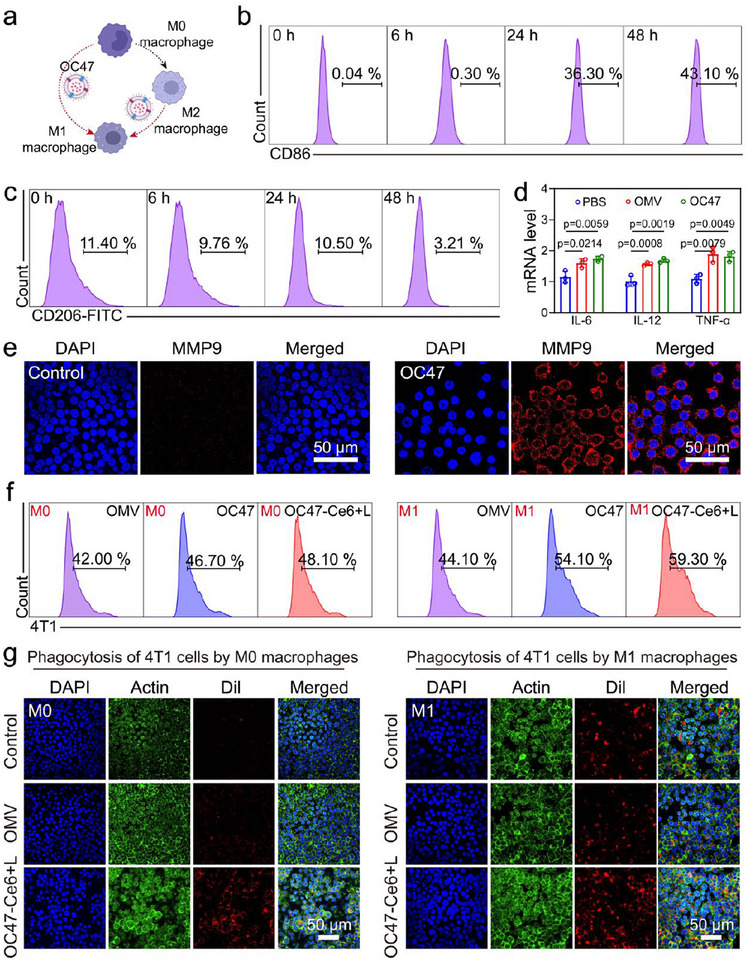
OC47 enhances M1 polarization and tumor cell phagocytosis. a) Schematic diagram of M1 polarization. b) Proportions of CD86^+^ cells in RAW 246.7 after OMV treatment at different times. c) Proportions of CD206^+^ cells in RAW 246.7 after OMV treatment at different times. d) mRNA levels of IL‐6, IL‐12, and TNF‐α in RAW 246.7 after different treatments. Data presented as mean ± SD, n = 3. e) Confocal image of RAW 246.7 expressing MMP9 after OC47 processing. Scale bar: 50 µm. f) Flow cytometry of engulfment of 4T1 cells by M0 and M1 macrophages after different treatments. g) Confocal images of M0 and M1 macrophages engulfing 4T1 cells after different treatments. Scale bar: 50 µm. The significance between each of the multiple groups in (d) was calculated using one‐way ANOVA. Figure 3a was created with BioRender.com released under a Creative Commons Attribution 4.0 International license (https://creativecommons.org/licenses/by/4.0/).

Next, we investigated whether OC47 could induce M1 polarization in macrophages (**Figure** **3**a). The proportion of CD86‐positive cells, a marker for M1‐type macrophages, was detected by flow cytometry. The results showed that with prolonged incubation of OC47 with M0‐type macrophages, the proportion of CD86‐positive cells gradually increased (Figure 3b). There was no significant difference between OMV and OC47 in increasing the proportion of CD86‐positive cells (Figure S5, Supporting Information). The proportion of CD206‐positive cells in M2 macrophages also decreased after co‐incubation with OC47 (Figure 3c). The qPCR results demonstrated that following the treatment of M0 macrophages with OC47, the expression of pro‐inflammatory cytokines, such as IL‐6, IL‐12, and TNF‐α, was significantly upregulated (Figure 3d). Furthermore, confocal microscopy revealed an increased expression of matrix metalloproteinase 9 (MMP9) on the surface of macrophages after OC47 treatment (Figure 3e). These findings suggested that OC47 modulated macrophage function by enhancing their pro‐inflammatory response. In summary, the results indicated that both OMV and OC47 possessed the ability to promote M1 polarization of macrophages and repolarization of M2 macrophages toward the M1 phenotype.

Subsequently, we investigated whether OC47 could interact with CD47 on the surface of tumor cells to block the “don't eat me” signal and enhance macrophage‐mediated phagocytosis of tumor cells. 4T1 cells labeled with Dil dye were treated with OMV, OC47, or OC47‐Ce6+L for 2 h, after which the treated 4T1 cells were co‐cultured with M0 or M1 macrophages. Flow cytometry results indicated that the proportion of 4T1 cells co‐cultured with macrophages increased over time in the OC47‐Ce6+L group, suggesting effective phagocytosis of 4T1 cells by M0 macrophages (Figure S6, Supporting Information). At the same time point, the number of 4T1 cells phagocytosed in the OC47 and OC47‐Ce6+L groups was higher than that in the OMV group (Figure 3f; Figure S7, Supporting Information). Furthermore, there was no significant difference in the phagocytosis efficacy between the OC47 and OC47‐Ce6+L groups, indicating that the introduction of Ce6 did not negatively impact the antitumor effect of OC47 in photodynamic therapy. Confocal microscopy revealed that 4T1 cells treated with OC47‐Ce6+L were more effectively phagocytosed by M1 macrophages (Figure 3g). Overall, these data suggested that engineering OC47‐Ce6 to block tumor cell CD47 could effectively disguise tumor cells as “pathogens,” facilitating their recognition and phagocytosis by macrophages.

### 2.3 Internalization of OC47‐Ce6 by NEs

**Figure 4:**
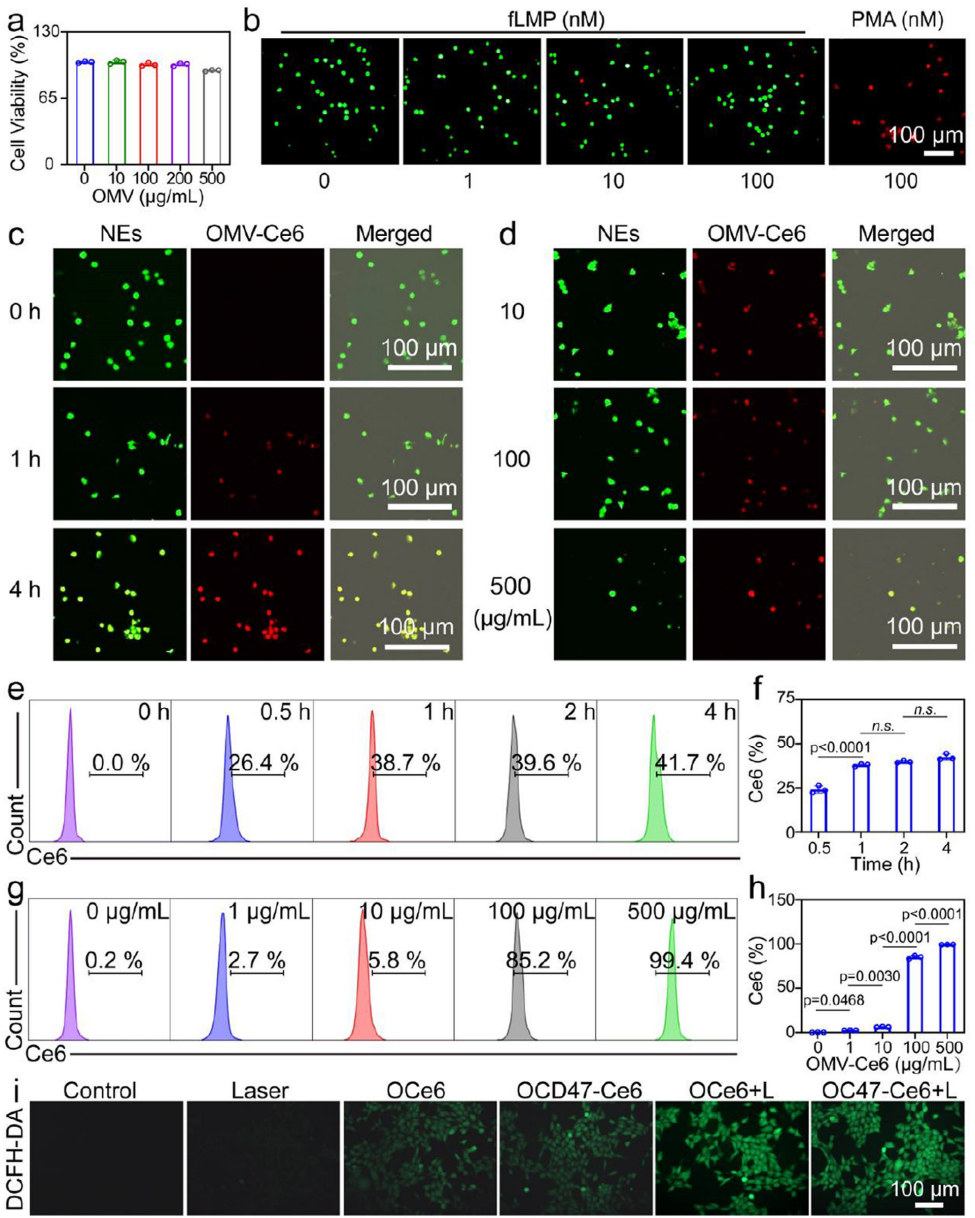
Internalization of OC47‐Ce6 by NEs. a) Cell viability rate of NEs. Data presented as mean ± SD, n = 3. b) Fluorescence imaging of calcineurin AM (green)/PI (red) staining in NEs treated with different concentrations of fLMP and PMA. Scale bar: 100 µm. c) Fluorescence imaging of OMV‐Ce6 co‐incubated with NEs for different durations. Scale bar: 100 µm. d) Fluorescence imaging of NEs co‐incubated with different concentrations of OMV‐Ce6. Scale bar: 100 µm. e, f) Flow cytometry and quantification of NEs co‐incubated with OMV‐Ce6 for different durations. Data presented as mean ± SD, n = 3. g, h) Flow cytometry and quantification of NEs co‐incubated with different concentrations of OMV‐Ce6. Data presented as mean ± SD, n = 3. i) Detection of ROS in 4T1 cells after different treatments. Scale bar: 100 µm. The significance between each of the multiple groups in (f) and (h) was calculated using one‐way ANOVA.

The effect of OC47‐Ce6 on the survival rate of NEs was evaluated using the CCK‐8 assay kit. The results indicated that even at high concentrations, OC47‐Ce6 did not have a significant impact on the survival rate of NEs (**Figure** **4**a). Propidium iodide staining results indicated that, compared to fMLP‐treated NEs, PMA‐treated NEs exhibited significant NETs formation (Figure 4b). Confocal microscopy (Figure 4c,d) and flow cytometry (Figure 4e–h) were used to evaluate the ability of NEs to load OMV‐Ce6. The experimental results showed that there were no significant differences in incubation times of 1, 2, and 4 h. The loading efficiency gradually increased with the increase in OMV‐Ce6 concentration. At a concentration of 500 µg mL^−1^, the positive peak reached 99.4%. Therefore, an incubation time of 1 h and a concentration of 500 µg mL^−1^ of OMV‐Ce6 were selected to ensure the maximum loading efficiency of OMV‐Ce6 with NEs. The ability of OC47‐Ce6 to generate ROS in 4T1 cells was evaluated using the DCFH‐DA ROS detection kit. The results showed that OC47‐Ce6 significantly increased ROS production within 4T1 cells, indicating its potential in photodynamic therapy (Figure 4i). Additionally, the CCK‐8 assay revealed that the combination of OC47‐Ce6 and laser irradiation significantly inhibited the proliferation of 4T1 cells (Figure S8, Supporting Information).

### 2.4 Antitumor Effects of NOC47‐Ce6

**Figure 5:**
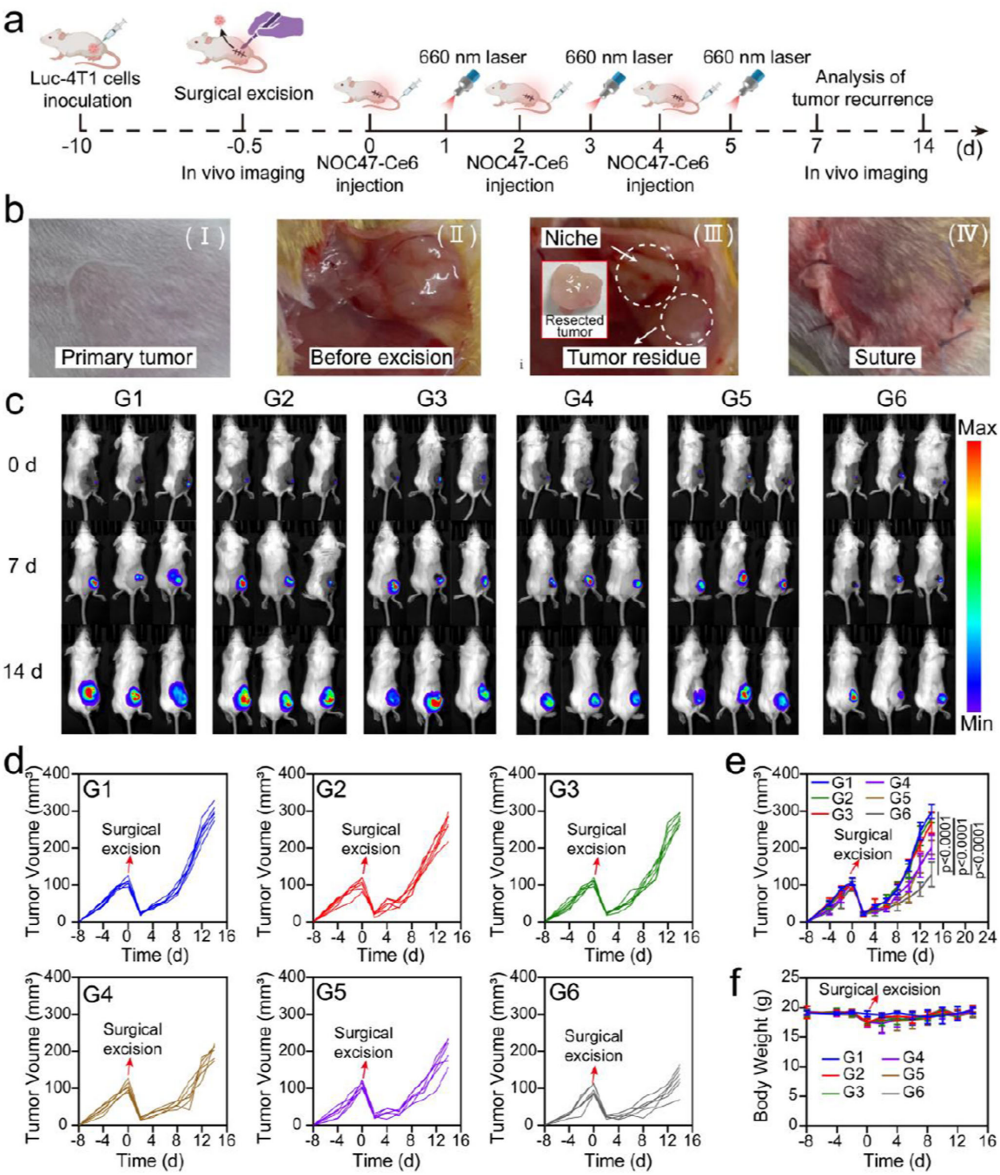
NOC47‐Ce6 inhibits tumor recurrence. a) Schematic diagram of surgical resection model of breast cancer in mice. b) Tumor excision process of breast cancer in mice. c) Chemiluminescence imaging of tumor sites in mice during treatment. d) Changes in tumor volume after different treatments. Data presented as mean ± SD, n = 6. e) Changes in tumor volume of each mouse after different treatments. f) Weight change curves of mice after different treatments. Data presented as mean ± SD, n = 6. (G1: PBS, G2: NEs+OMV, G3: OMV‐Ce6+Laser, G4: NOC47‐Ce6, G5: NO‐Ce6+Laser, G6: NOC47‐Ce6+Laser). The significance between each of the multiple groups in (e) was calculated using one‐way ANOVA. Figure 5a was created with BioRender.com released under a Creative Commons Attribution 4.0 International license (https://creativecommons.org/licenses/by/4.0/).

**Figure 6:**
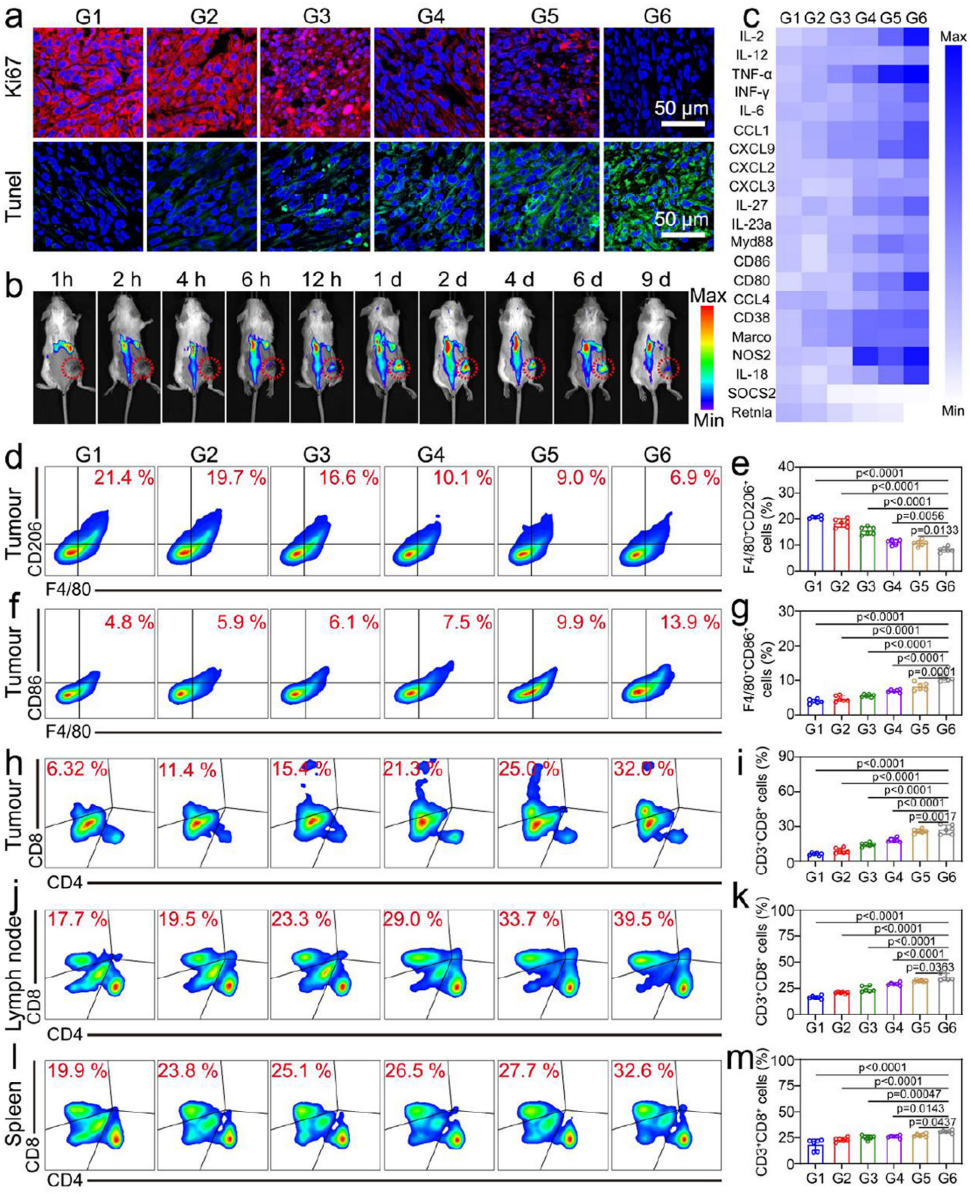
Immune activation of NO47‐Ce6. a) Ki67 and Tunel staining of tumors. Scale bar: 50 µm. b) NEs targeted tumor resection site. c) mRNA levels of inflammatory factors in tumors after different treatments, n = 3. Flow cytometry and quantitative analysis of CD206^+^F4/80^+^ cells (d, e) and CD86^+^F4/80^+^ cells (f, g) in tumors. Data presented as mean ± SD, n = 6. Flow cytometry and quantitative analysis of CD8^+^CD3^+^cells in tumors (h, i), lymph nodes (j, k), and spleen (l, m). Data presented as mean ± SD, n = 6. (G1: PBS, G2: NEs+OMV, G3: OMV‐Ce6+Laser, G4: NOC47‐Ce6, G5: NO‐Ce6+Laser, G6: NOC47‐Ce6+Laser). The gating strategy for flow cytometry analysis is used in Figures 6d–m (Figure S14, Supporting Information). The significance between each of the multiple groups in (e, g, i, k, m) was calculated using one‐way ANOVA.

To evaluate the antitumor efficacy of NOC47‐Ce6, we employed a partially resected tumor tissue mouse model and examined the suppression of postoperative tumor recurrence (**Figure** **5**a). To simulate the clinical scenario of incomplete tumor resection, 4T1‐Luc breast cancer cells were implanted into the dorsal region of mice. When the tumor volume reached 120 mm^3^, 90% of the tumor tissue was surgically excised, followed by suturing (Figure 5b). Mice were randomly assigned to different treatment groups: PBS, NEs+OMV, OMV+Laser, NOMV‐Ce6+Laser, NOC47‐Ce6, and NOC47‐Ce6+Laser. The OMV+Laser, NOMV‐Ce6+Laser, and NOC47‐Ce6+Laser groups received 660 nm laser irradiation on days 1, 3, and 5. Bioluminescent signals were recorded to monitor the regrowth of tumor remnants. On the 14th day post‐surgery, no significant differences in bioluminescence were observed among the PBS, NEs+OMV, and OMV+Laser groups of mice. However, the NOMV‐Ce6+Laser, NOC47‐Ce6, and NOC47‐Ce6+Laser groups exhibited significantly weaker bioluminescent signals (Figure 5c). The NOC47‐Ce6+Laser group demonstrated the most effective tumor inhibition. Concurrently, we measured the direct volume changes of recurrent tumors, which were consistent with the bioluminescence analysis results (Figure 5d,e). Throughout the treatment process, no significant changes in body weight were observed in the mice (Figure 5f). Additionally, tumor tissues were examined using terminal deoxynucleotidyl transferase dUTP nick end labeling (Tunel), and Ki‐67 immunofluorescence staining. There was a significant reduction in red fluorescence of Ki‐67 and a significant increase in green fluorescence of Tunel staining, further confirming the high efficacy of the combined treatment in inhibiting tumor growth (**Figure** **6**a). The hemolysis experiment was conducted to assess the biocompatibility of NOC47‐Ce6 by examining its in vitro hemolytic effect on red blood cells. The results showed that after co‐incubation of NEs, OC47‐Ce6, and NOC47‐Ce6 with red blood cells for 3 h, the hemolysis rate was less than 1% for all groups (Figure S9, Supporting Information). In order to further evaluate the biocompatibility of the application of NOC47‐Ce6+Laser in vivo, complete blood count (CBC) and serum biochemistry were performed after the end of NOC47‐Ce6+Laser treatment. The results indicated no significant changes in white blood cells (WBC), red blood cells (RBC), hemoglobin (HGB), platelets (PLT), alanine aminotransferase (ALT), aspartate aminotransferase (AST), Serum Creatinine (CREA), and UREA (Figure S10, Supporting Information). Histological analysis of the heart, liver, spleen, lungs, and kidneys using H&E staining also showed no noticeable changes. These findings demonstrated that NOC47‐Ce6+Laser exhibited favorable biosafety in vivo (Figure S11, Supporting Information). NEs were isolated from the peripheral blood of mice and labeled with DIR dye. These labeled NEs were then intravenously injected via the tail vein into mice with a surgical resection model. Using a small animal imaging system, the targeting of NEs to the surgical site was observed. The results indicated that NEs accumulated most abundantly at the surgical site 24 h post‐injection, demonstrating their significant targeting capability to the surgical site (Figure 6b).

### 2.5 NOC47‐Ce6 Activates an Anti‐Tumor Immune Response

Quantitative analysis of M1‐associated markers in tumor tissue using qPCR. We found that treatment with NOC47‐Ce6+Laser significantly upregulated various M1‐associated markers in the tumor tissues, including CD80, CD86, and IL family cytokines. Notably, the increase in certain M1‐associated chemokines might contribute to the recruitment of immune cells, thereby enhancing the immune response to the treatment. In contrast, M2‐associated markers were reduced (Figure 6c). Further investigation into the polarization state of TAMs following NOC47‐Ce6 treatment was conducted. Flow cytometry analysis revealed that the proportion of M2 macrophage markers (F4/80^+^CD206^+^ cells) in the NOC47‐Ce6+Laser group was significantly reduced, while the proportion of M1 macrophage markers (F4/80^+^CD86^+^ cells) was significantly increased compared to the PBS group (Figure 6d–g). This indicated that NOC47‐Ce6+Laser treatment effectively induced the polarization of macrophages toward the pro‐inflammatory M1 phenotype and inhibited the formation of the anti‐inflammatory M2 phenotype. This shift in polarization not only enhanced the local immune response but also contributed to the remodeling of the tumor microenvironment. Subsequently, we examined the changes in CD8^+^ T cells in the tumors, spleens, and lymph nodes of mice after three treatments. The percentages of CD8^+^ T cells in the spleen, lymph nodes, and tumors of the NOC47‐Ce6+Laser group were 12.7%, 21.8%, and 25.7% higher, respectively, compared to the PBS group. Additionally, the percentages of CD8^+^ T cells in the spleen, lymph nodes, and tumors of the NOC47‐Ce6+Laser group were 6.1%, 10.5%, and 10.7% higher, respectively, than those in the NOC47‐Ce6 group. This demonstrated that the combination of ROS and aCD47 exerted a more potent immunostimulatory effect (Figure 6h–m).

**Figure 7:**
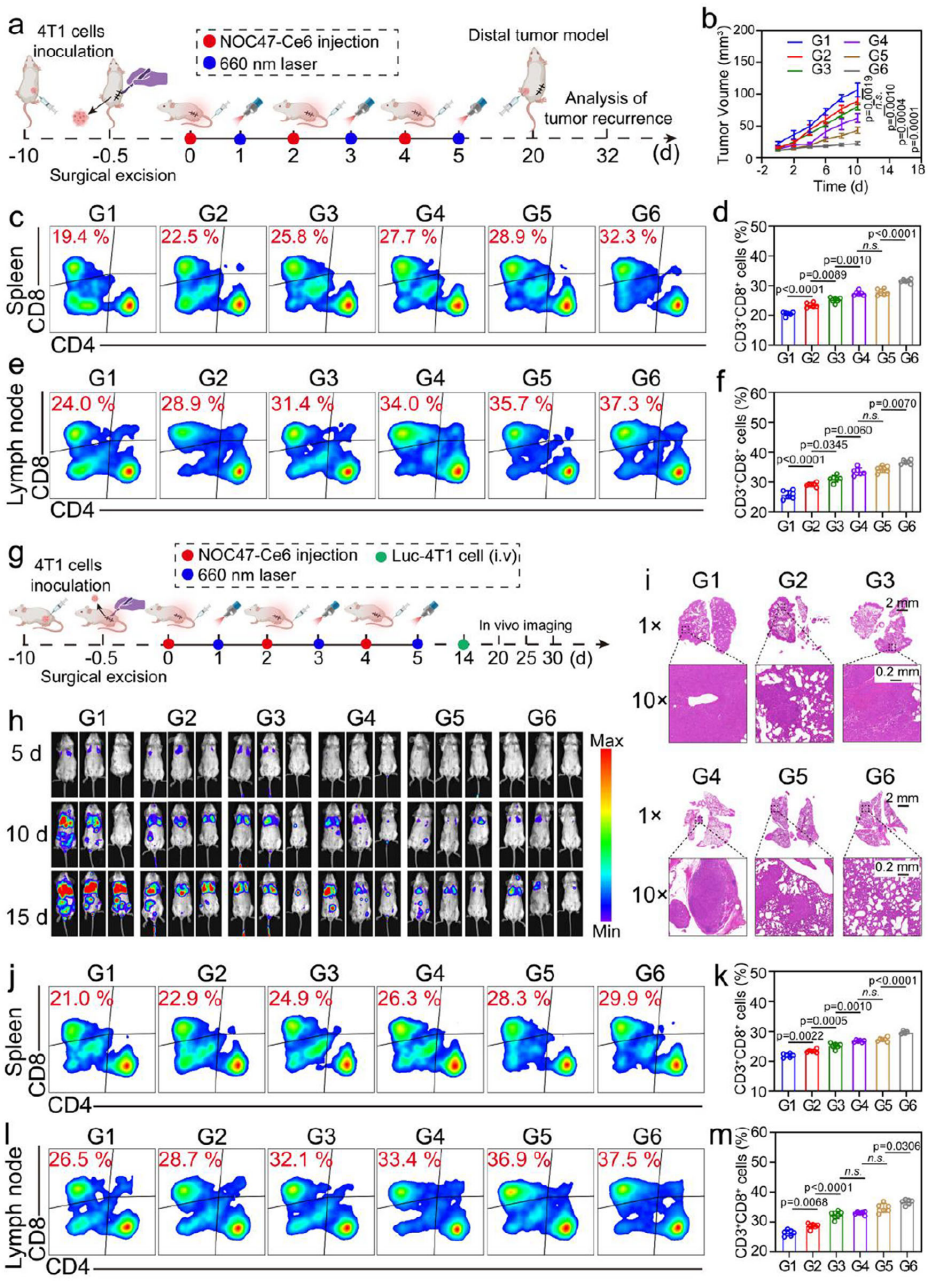
NOC47‐Ce6 activates long‐term immune effects. a) Schematic diagram of a mouse distal tumor model. b) Changes in tumor volume on the contralateral side of mice after different treatments. Data presented as mean ± SD, n = 6. Flow cytometry and quantitative analysis of CD8^+^CD3^+^cells in the spleen (c, d), and lymph nodes (e, f). Data presented as mean ± SD, n = 6. g) Schematic diagram of mouse lung metastasis model. h) Bioluminescence imaging of tumors in metastatic model mice. i) Representative images of H&E‐stained lung tissue.Scale bar: 2 mm (top) and 0.2 mm (bottom). Flow cytometry and quantitative analysis of CD8^+^CD3^+^cells in the spleen (j, k), and lymph nodes (l, m). Data presented as mean ± SD, n = 6. (G1: PBS, G2: NEs+OMV, G3: OMV‐Ce6+Laser, G4: NOC47‐Ce6, G5: NO‐Ce6+Laser, G6: NOC47‐Ce6+Laser). The gating strategy for flow cytometry analysis is used in Figures 7e–h, k–n (Figure S14, Supporting Information). The significance between each of the multiple groups in (b, d, f, k, m) was calculated using one‐way ANOVA. Figure 7a,g as created with BioRender.com and released under a Creative Commons Attribution 4.0 International license (https://creativecommons.org/licenses/by/4.0/).

To validate the activation of immune memory by NOC47‐Ce6, we established two animal models. In the first model, 4T1 cells were subcutaneously injected into the contralateral side on the 20th day of treatment, and tumor growth was monitored (**Figure** **7**a). The results showed that mice receiving NOC47‐Ce6+Laser treatment exhibited slow growth of subcutaneous 4T1 tumors and no significant changes in body weight (Figure 7b; Figure S12, Supporting Information). Flow cytometry analysis revealed that the proportion of CD8^+^ T cells in the spleen and lymph nodes of the NOC47‐Ce6+Laser group was 12.9% and 13.3% higher, respectively, compared to the PBS group (Figure 7c–f). In the second model, we constructed a lung metastasis model using 4T1 cells transfected with a luciferase reporter gene. The bioluminescence in lung tissues was recorded to analyze the anti‐metastatic effects of different treatment groups (Figure 7g). The results illustrated that the bioluminescence signal in the lung tissues of the PBS, NEs+OMV, and OMV+Laser groups increased over time. The signals in the NOMV‐Ce6+Laser and NOC47‐Ce6 groups were relatively weaker, with the NOC47‐Ce6+Laser group showing the weakest bioluminescence signal (Figure 7h). H&E staining of the lung revealed that the NOC47‐Ce6+Laser group had the fewest metastatic nodules (Figure 7i; Figure S13, Supporting Information). Flow cytometry analysis demonstrated that the proportion of CD8^+^ T cells in the spleen and lymph nodes of the NOC47‐Ce6+Laser group was 8.9% and 11.0% higher, respectively, compared to the PBS group. These findings suggested that mice in the NOC47‐Ce6+Laser group exhibited stronger anti‐tumor responses and more durable immune memory when rechallenged with tumor cells (Figure 7j–m).

## 3 Conclusion

In summary, we utilized OC47‐Ce6 hitchhiking on NEs to achieve precise targeted release at the surgical resection site. By reprogramming TAMs and implementing immune checkpoint blockade, the phagocytic function of TAMs was activated, demonstrating the potential to suppress postoperative recurrence and metastasis in breast cancer. Experimental results indicated that the combination of NC47‐Ce6 polarization and CD47 blockade synergistically enhanced TAM phagocytosis. Additionally, the photosensitizing properties of Ce6 further increased tumor cell killing efficacy, promoting the release of abundant tumor‐associated antigens, thereby activating a T cell‐mediated antitumor immune response.

## 4 Experimental Section

### Cell Lines

Mouse breast cancer cells (4T1) and mouse breast cancer cells‐luciferase labeled (4T1‐LUC) were obtained from Wuhan Hualianke Biotechnology Co., Ltd. Mouse mononuclear macrophage leukemia cells (RAW264.7) were obtained from Wuhan Saitekang Biotechnology Co., Ltd. All three cells were cultured in DMEM medium containing 10% fetal bovine serum, 100 U mL^−1^ penicillin and 0.1 mg mL^−1^ streptomycin.

### Animals

Balb/c mice (6–8 weeks old, weighing 18–20 g) were obtained from Hunan Slyke Jingda Laboratory Animal Co., Ltd. The mice were maintained in a controlled animal facility with stable environmental conditions (temperature: 22±1 °C, relative humidity: 40%–70%, and a 12 h light–dark cycle), with ad libitum access to food and water. All animal procedures were approved by the Animal Experimentation Ethics Committee of Huazhong University of Science and Technology (IACUC Number: 4208). Tumor volume formula: volume = (length × width^2^)/2. To minimize animal discomfort, according to the Guideline of Assessment for Humane Endpoints in Animal Experiments (Certification and Accreditation Administration of the P. R. China, RB/T 173–2018), in general experiments, the tumor burden should not exceed 5% of the animal's normal body weight; in therapeutic experiments, it should not exceed 10% of the animal's body weight (10% indicated that the diameter of the subcutaneous tumor on the back of a 25 g mouse reached 17 mm). At the end of the mouse experiments, mice were euthanized according to animal welfare standards.

### Extraction of OMV

MG1655 was cultured on solid LB agar plates at 37 °C for 12 h. A single colony was then inoculated into an LB liquid medium and incubated at 37 °C in a shaking incubator until the optical density (OD) reached 1.0. The 1L bacterial culture was centrifuged at 8000 g for 20 min to remove the bacteria, and the supernatant was filtered through a 0.45 µm vacuum filter. The filtrate was concentrated using a centrifugal filter with a 100 kDa molecular weight cutoff. The concentrated culture medium was then centrifuged at 200000 g for 3 h at 4 °C. The OMV pellet was resuspended in PBS and filtered through a 0.22 µm vacuum filter to prevent bacterial contamination. The extraction method for OC47 was the same as the method for OMV.

### Preparation of OCe6 and OC47‐Ce6

150 µg of OMV and OMV‐CD47 were each mixed with 30 µg of Ce6 and incubated at 4 °C for 2 h with gentle stirring. The mixture was then subjected to repeat washing using a centrifugal filter with a 100 kDa molecular weight cutoff, followed by washing with PBS until the unbound Ce6 was completely removed. The resulting preparations were OCe6 and OC47‐Ce6.

### In Vivo Antitumor Efficacy

A subcutaneous tumor model was established by injecting ≈1×10^6^ 4T1 cells into the right flank of Balb/c mice. When the tumor reached 100 mm^3^ in size, ≈90% of the tumor tissue was surgically excised. After surgery, the mice were randomly divided into six groups: PBS, NEs+OMV, OMV‐Ce6+Laser, NOC47‐Ce6, NO‐Ce6+Laser, and NOC47‐Ce6+Laser. The treatment doses for each group were as follows: NEs: 2×10^7^ particles per mouse, OMV: 50 µg per mouse, CD47: 4.5 µg per mouse, Ce6: 5 µg per mouse. 24 h after injection, the laser‐treated groups were exposed to a 660 nm wavelength laser at a power density of 5 mW cm^−2^ for 10 min. During the treatment period, body weight and tumor volume changes were recorded every other day. Tumor recurrence was monitored using in vivo fluorescence imaging. D‐Luciferin potassium salt was dissolved in PBS to prepare a 15 mg mL^−1^ solution, which was subsequently sterilized by filtration through a 0.2 µm membrane filter. The solution was administered to mice via intraperitoneal injection at a dose of 10 µL g^−1^ of body weight. Each mouse (20 g) received an intraperitoneal injection of 200 µL (equivalent to 3 mg of D‐luciferin potassium salt). After a 15 min incubation period to allow systemic distribution, in vivo imaging analysis was performed using an appropriate imaging system. On day 8 post‐surgery, three mice from each group were sacrificed. Tumor, spleen, and lymph nodes were collected and processed into single‐cell suspensions. After incubation with the appropriate antibodies, flow cytometry was used to analyze the proportions of F4/80^+^CD206^+^ cells, F4/80^+^CD86^+^ cells, CD3^+^CD4^+^ T cells, and CD3^+^CD8^+^ T cells in the tumor, spleen, and lymph nodes. All flow cytometry data were analyzed using FlowJo software.

Additional experimental details are described in Supporting Information.

### Statistical Analysis

Data are expressed as mean ± S.D. Significance between two groups was assessed by unpaired two‐tailed Student's t‐test and between each of the multiple groups was calculated using one‐way ANOVA. Values with *P* < 0.05 were considered significant. Exact p values were provided accordingly in the figures. All the statistical analyses were performed using GraphPad Prism (8.0). Flow cytometry data were analyzed with FlowJo (ver. 10.8.1). Confocal images were analyzed with FluoView31S (ver.2.3.1.163).

## Conflict of Interest

The authors declare no conflict of interest.

## Supporting information



Supporting Information

## Data Availability

The raw/processed data required to reproduce these findings cannot be shared at this time as the data also forms part of an ongoing study.
