# Supplementary material for: Engineered Bacterial Outer Membrane Vesicles Hitchhiking on Neutrophils for Antibody Drug Delivery to Enhance Postoperative Immune Checkpoint Therapy
Source: Adv Sci (Weinh). 2025 Apr 27;12(27):2505000. doi: 10.1002/advs.202505000 (PMC12279201; doi:10.1002/advs.202505000)
Supplement: Supplementary file 1 — Supporting Information [file ADVS-12-2505000-s001.pdf]

ADVANCED SCIENCE
Open Access

Supporting Information

for *Adv. Sci.*, DOI 10.1002/advs.202505000

Engineered Bacterial Outer Membrane Vesicles Hitchhiking on Neutrophils for Antibody Drug Delivery to Enhance Postoperative Immune Checkpoint Therapy

Meng Guan, Xiao-Ting Xie, Dong Zhou, Kai Cheng, Bin Zhang, Xin-Yue Xu, Yong Li, Yi-Tong Zhou, Wei Peng, Li-Li Chen, Peng-Shuo Dong, Si Chen, Jia-Hua Zou, Bo Liu*, Yuan-Di Zhao* and Jin-Xuan Fan**

Supporting Information

of

Engineered Bacterial Outer Membrane Vesicles Hitchhiking on Neutrophils for Antibody Drug Delivery to Enhance Postoperative Immune Checkpoint Therapy

Meng Guan, Xiao-Ting Xie, Dong Zhou, Kai Cheng, Bin Zhang, Xin-Yue Xu, Yong Li, Yi-Tong Zhou, Wei Peng, Li-Li Chen, Peng-Shuo Dong, Si Chen, Jia-Hua Zou, Bo Liu*, Yuan-Di Zhao*, Jin-Xuan Fan**

M. Guan, X.-T. Xie, K. Cheng, B. Zhang, X.-Y. Xu, Y. Li, Y.-T. Zhou, L.-L. Chen, P.-S. Dong, J.-H. Zou, B. Liu, Y.-D. Zhao, J.-X. Fan
Britton Chance Center for Biomedical Photonics at Wuhan National Laboratory for Optoelectronics - Hubei Bioinformatics & Molecular Imaging Key Laboratory, Department of Biomedical Engineering, College of Life Science and Technology, Huazhong University of Science and Technology, Wuhan 430074, Hubei, P. R. China

D. Zhou, J.-H. Zou
Department of Oncology, Huanggang Central Hospital of Yangtze University, Huanggang 438000, Hubei, P. R. China

D. Zhou, W. Peng, J.-H. Zou
Hubei Clinical Medical Research Center of Esophageal and Gastric Malignancy, Huanggang City 438021, Hubei, P.R. China

S. Chen
Hubei Key Laboratory of Plasma Chemistry and Advanced Materials, School of Material Science and Engineering, Wuhan Institute of Technology, Wuhan 430205, Hubei, P. R. China

Y.-D. Zhao
Key Laboratory of Biomedical Photonics (HUST), Ministry of Education, Huazhong University of Science and Technology, Wuhan 430074, Hubei, P. R. China

Keywords: Engineered bacterial; Outer membrane vesicles; Neutrophils; Immunotherapy

Corresponding author: zoujiahua@hgyy.org.cn (J.H. Zou); lbyang@mail.hust.edu.cn (B. Liu); zydi@mail.hust.edu.cn (Y.D. Zhao); jxfan@hust.edu.cn (J.X. Fan)

Methods

Materials

DiR and CD47 protein were purchased from MedChemExpress (New Jersey, USA). The gene sequence of anti-CD47 peptide (aCD47) was obtained from NCBI and synthesized by Miaoling Biotechnology Co., Ltd. (Wuhan, China). The CD47 Enzyme-Linked Immunosorbent Assay (ELISA) kit was purchased from Bioswap Life Science Laboratory (Wuhan, China). Fetal bovine serum (FBS) was purchased from Zhejiang Tianhang Biotechnology Co., Ltd. (Zhejiang, China). Trypsin, PBS, and DMEM were purchased from Servicebio Biotechnology Co., Ltd. (Wuhan, China). Cell Counting Kit-8, 1,1'-Dioctadecyl-3,3,3'-tetramethylindocarbocyanine (DiI), 4',6-diamidino-2-phenylindole (DAPI), BCA Protein Assay Kit, Calcein Acetoxymethyl Ester (Calcein AM), Propidium Iodide (PI), Reactive Oxygen Species (ROS) Assay Kit, SDS-PAGE sample loading buffer (5X), hydrophobic polyvinylidene fluoride (PVDF) membrane, penicillin-streptomycin, RNase-free dd-H_2O, 4% paraformaldehyde fixative, and Tween-20 were purchased from Shanghai Beyotime Biotechnology Co., Ltd. (Shanghai, China). Sodium hydroxide, absolute ethanol, dimethyl sulfoxide (DMSO), isopropanol, sodium chloride, sodium dodecyl sulfate (SDS), anhydrous methanol, concentrated nitric acid, and hydrochloric acid were purchased from Sinopharm Chemical Reagent Co., Ltd. (Shanghai, China). RNA isolation reagent, HiScript III All-in-one RT SuperMix Perfect for qPCR kit, and Taq Pro Universal SYBR qPCR Master Mix kit were purchased from Vazyme Biotech Co., Ltd. (Nanjing, China). ECL Chemiluminescence Substrate Kit was purchased from Beijing Labgic Technology Co., Ltd. (Beijing, China). Omni-Easy™ One-Step PAGE Gel Quick Preparation Kit was purchased from Shanghai Yaenzyme Biopharmaceutical Technology (Shanghai, China). Phenylmethanesulfonyl fluoride (PMSF), Tris(hydroxymethyl)aminomethane (Tris), and glycine were purchased from Beijing Kehbio Techology Co., Ltd. (Beijing, China). All solutions were prepared using ultra-pure water from the Milli-Q system ($\geq$ 18.20 M Ω). The antibody information is as follows: APC anti-mouse CD4 Antibody (BioLegend, Cat. No.

100411, Clone: GK1.5, diluted to 1:100); FITC anti-mouse CD8 Antibody (BioLegend, Cat. No. 980908, Clone: SK1, diluted to 1:100); PE anti-mouse CD3 Antibody (BioLegend, Cat. No. 100205, Clone: 17A2, diluted to 1:100); FITC anti-mouse CD86 Antibody (BioLegend, Cat. No. A17199A, Clone: 159219, diluted to 1:100); PE anti-mouse CD206 Antibody, (BioLegend, Cat. No. 141706, Clone: C068C2, diluted to 1:100); APC anti-mouse F4/80 Antibody (BioLegend, Cat. No. 123116, Clone: BM8, diluted to 1:100); Cy5 goat anti rabbit IgG (Service, Cat. No. GB27303, diluted to 1:200); FITC goat anti rabbit IgG (Service, Cat. No. GB22303, diluted to 1:200).

Extraction of NEs

NEs were isolated from mouse blood using a neutrophil isolation kit for mouse peripheral blood (purchased from Tianjin Haoyang Huake Biotechnology Co., Ltd.).

Preparation of NOMV-Ce6 and NOC47-Ce6

NOMV-Ce6 and NOC47-Ce6 were obtained by incubating the purified NEs with OMV-Ce6/OC47-Ce6 at 4°C for 1 h, followed by collection through centrifugation.

Release of OC47-Ce6

Treating NOC47-Ce6 with PBS, fMLP, and PMA for different durations, collecting the supernatant after centrifugation, and determining the Ce6 content using UV visible absorption spectroscopy to calculate the loading amount of OC47-Ce6 in NOC47-Ce6.

SDS-PAGE gel electrophoresis

The separation gel and stacking gel are prepared and allowed to polymerize. The protein samples are then mixed with sample buffer, heated at 95°C for denaturation, and loaded onto the gel. Electrophoresis was performed at 80-120 V until the dye front approached the bottom of the gel. After electrophoresis, the gel was stained with Coomassie Brilliant Blue and destained to remove background staining. Finally, the protein banded on the gel were observed and analyzed by comparing them to the molecular weight marker to determine the protein profile of the sample.

RNA extraction

After different treatments, washing the adherent cells three times with PBS and adding 1 mL of RNA isolation reagent. Gently pipetting the cells and collect them into a centrifuge tube. Then, adding 200 μL of chloroform, vigorously shaking for 15 seconds to form an emulsion, and incubating on ice for 5 min. Afterwards, centrifuging at 12,000 g for 15 min at 4°C. Transferring the upper aqueous phase to a new centrifuge tube and adding an equal volume of pre-chilled isopropanol. Gently inverting to mix and incubating on ice for 10 min. Centrifuging again at 12,000 g for 10 min at 4°C, during which a white pellet should typically be visible. After removing the supernatant, adding 1 mL of 75% ethanol, inverting a few times, and incubating at room temperature for 3-5 min. Then, centrifuging at 12,000 g for 5 min at 4°C and discarding the supernatant. Drying the pellet for 2-5 min, then adding an appropriate amount of RNase-free ddH_2O to dissolve the RNA. Finally, using a micro spectrophotometer to measure the A_{206}/A_{230} and A_{260}/A_{280} ratios.

Detection of M1 and M2 macrophage markers

RAW267.4 cells were treated with OMV, OC47, or OC47-Ce6+Laser (10 μg OMV/mL) for different time points, and then flow cytometry was used to assess M1 macrophage markers (F4/80$^+$CD86$^+$ cells) and M2 macrophage markers (F4/80$^+$CD206$^+$ cells).

Cell immunofluorescence assay

Cells were seeded onto coverslips in a 24-well plate and treated with OC47 for 48 h. After treatment, the cells were washed three times with PBS. They were then fixed with 4% paraformaldehyde for 20 min. Following fixation, the cells were permeabilized with permeabilization buffer for 20 min to enhance antibody penetration. Next, the cells were blocked with 5% BSA at room temperature for 1 h to prevent nonspecific binding. Each well was then incubated with 50 μL of primary antibody at room temperature for 1 h. After incubation, the cells were washed three times with PBS. The secondary antibody was added (50 μL per well), and incubation continued at room temperature for 1 h. Finally, after three washing

with PBS, the cells were mounted using an anti-fade mounting medium containing DAPI for nuclear staining.

Cytotoxicity of OMV towards NEs

The isolated NEs were added to a 96-well plate, with approximately 10^5 cells per well. After co-incubating the NEs with different concentrations of OMV for 2 h, CCK-8 reagent was added to assess the cell viability of the NEs.

Detection of Ce6 in NOC47-Ce6

After co-incubating NEs with different concentrations of OC47-Ce6 for varying time points, unbound OC47-Ce6 was removed by centrifugation. The fluorescence signal of Ce6 was then detected using confocal fluorescence microscopy and flow cytometry.

In vivo safety evaluation

To assess the biocompatibility of NOC47-Ce6+Laser, blood samples were collected from mice at the end of the treatment. A portion of 300 μL of blood was transferred into a coagulation tube containing separation gel and centrifuged at 10,000 rpm for 10 min. The resulting supernatant was used for liver and kidney function analysis, measuring levels of AST, ALT, CREA, and UREA. Additionally, 100 μL of blood was collected into an anticoagulant tube for routine blood tests, including RBC, WBC, PLT, and HGB measurements. Furthermore, the heart, liver, spleen, lungs, and kidneys of the mice were harvested and weighed. The tissues were then fixed in 4% paraformaldehyde, followed by dehydration, embedding, and sectioning. Finally, H&E-stained tissue sections were examined under an optical microscope for histopathological analysis.

Supplementary Figures

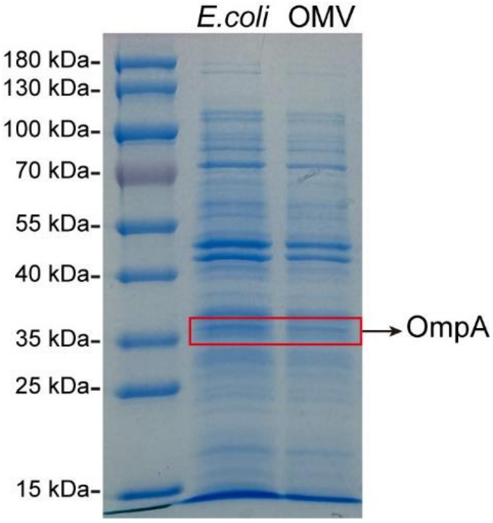

Figure S1 SDS-PAGE of the *E. coli* and OMV.

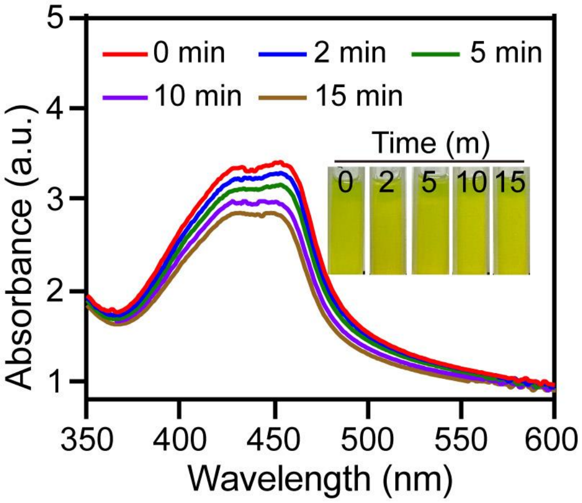

Figure S2. UV-vis absorption spectra of DPBF solution after irradiation with 606 nm laser for different durations. The inset shows a white light image of the color change in DPBF.

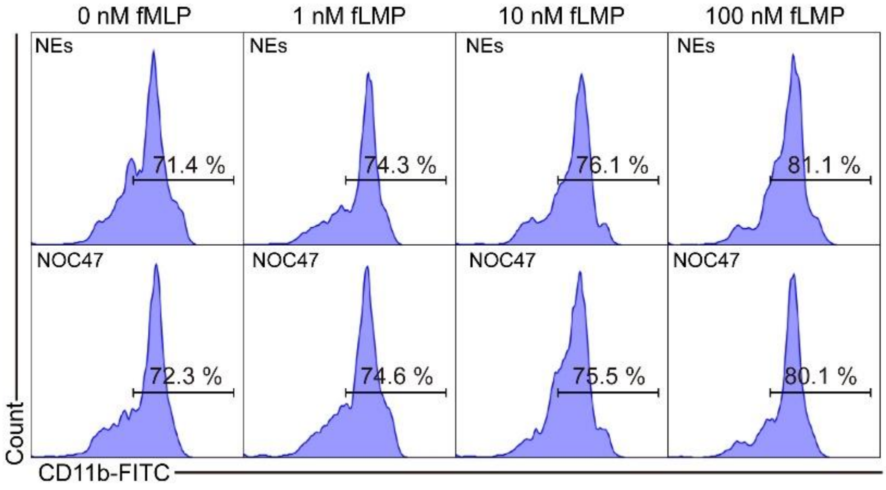

Figure S3 Changes in CD11b expression levels on NEs and NOC47 cell membrane after treatment with different concentrations of fMLP for 1 h.

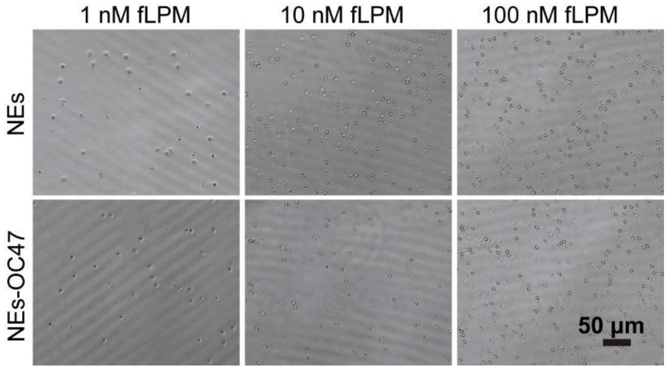

Figure S4 Images of NEs migration in the lower chamber of a transwell system under various fMLP concentrations. Scale bar, 50 μm.

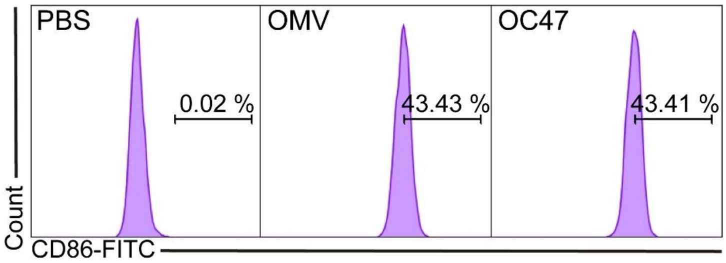

Figure S5 Proportions of $CD86^+$ cells in RAW 264.7 macrophages after 4 h of treatment with OMV and OC47.

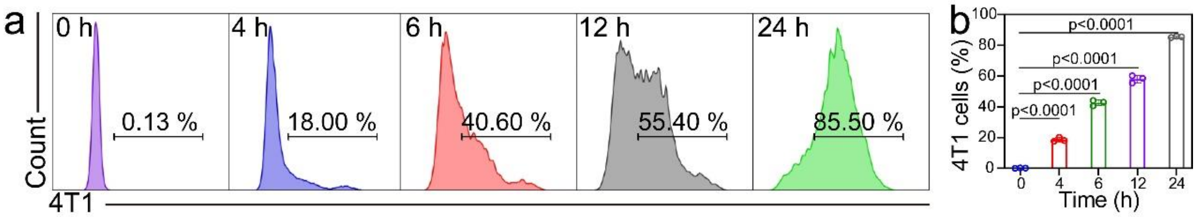

Figure S6 Flow cytometry analysis (**a**) and quantification (**b**) of 4T1 cells incubated with M1 macrophages for different durations after treatment with OC47-Ce6+L. Data presented as mean ± SD, n=3. Significance between each of the multiple groups in (**b**) was calculated using one-way ANOVA.

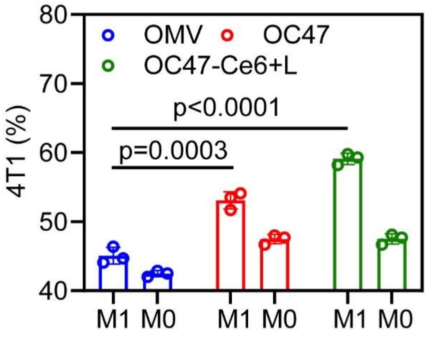

Figure S7 Quantitative analysis of the phagocytosis of 4T1 cells by M0 and M1 macrophages after different treatments using flow cytometry. Data presented as mean ± SD, n=3. Significance between each of the multiple groups was calculated using one-way ANOVA.

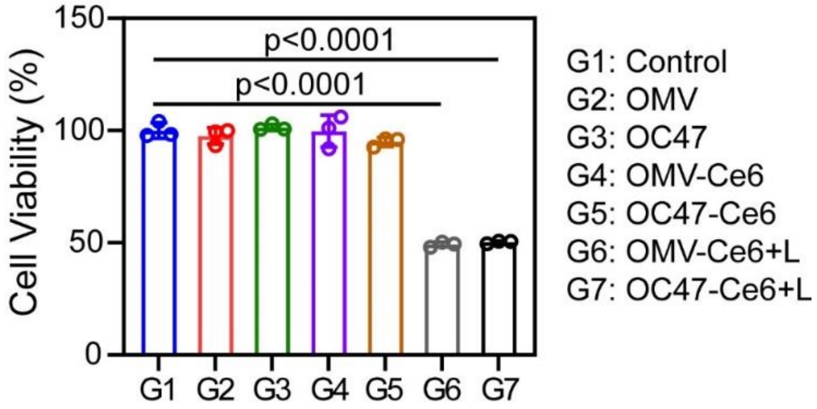

Figure S8 Cell viability of 4T1 cells after different treatments. Data presented as mean ± SD, n=3. Significance between each of the multiple groups was calculated using one-way ANOVA.

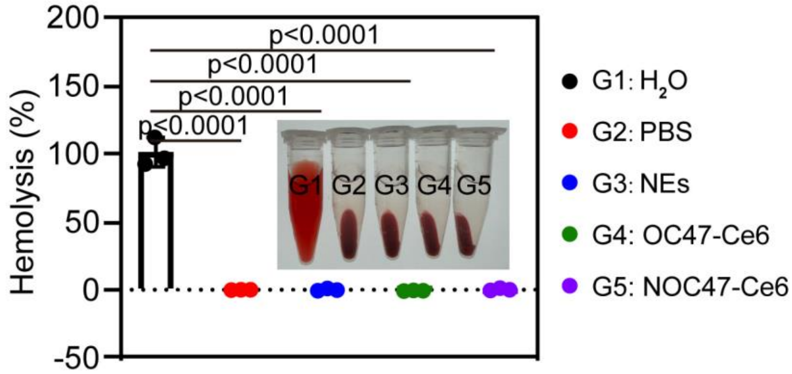

Figure S9 Bright field plot (inset) and hemolysis rate of NEs, OC47-Ce6, and NC47-Ce6 cultured erythrocytes. Data presented as mean ± SD, n=3. Significance between each of the multiple groups was calculated using one-way ANOVA.

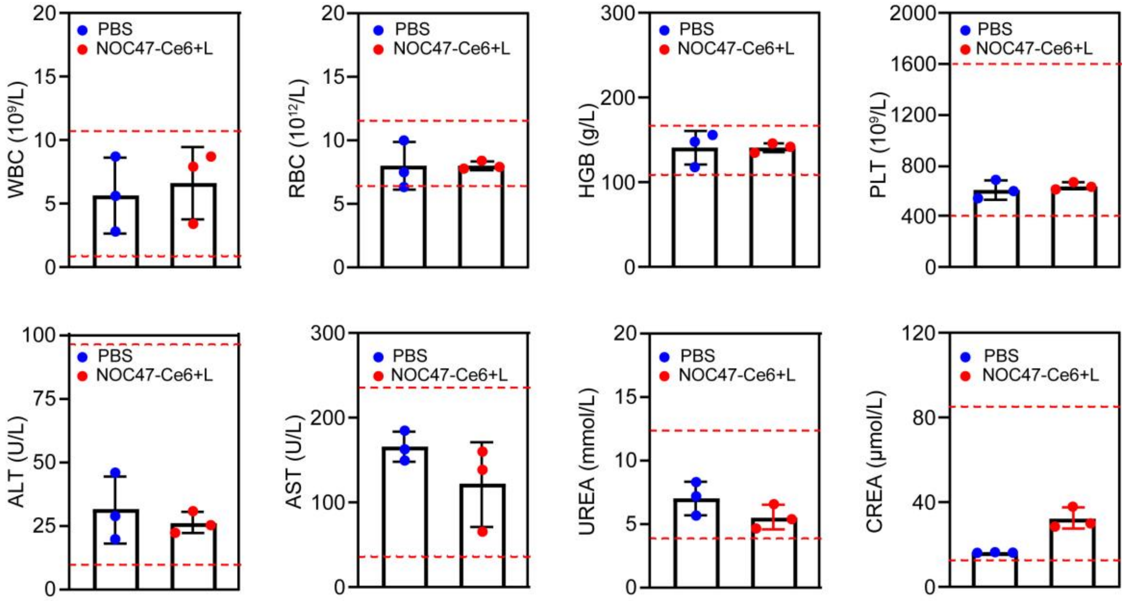

Figure S10 Complete blood count and serum biochemistry examination indicators of mice after different treatments (the red dotted line represents the normal range). Data presented as mean ± SD, n=3.

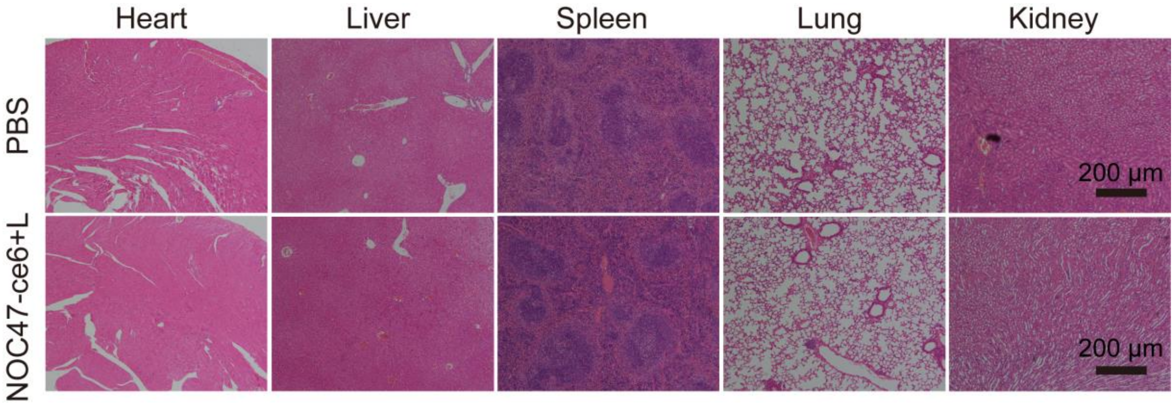

Figure S11 H&E staining results of heart, liver, spleen, lung and kidney of mice after different treatments. Scale bar: 200 μm.

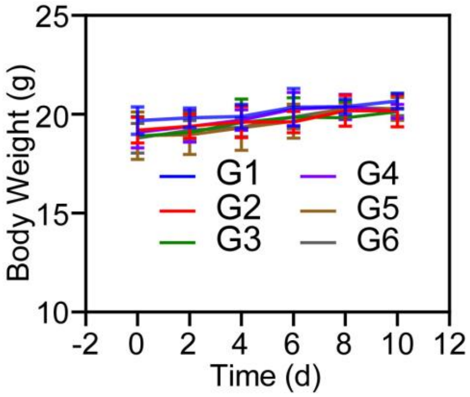

Figure S12 Weight change curves of mice after different treatments. Data presented as mean ± SD, n=6.

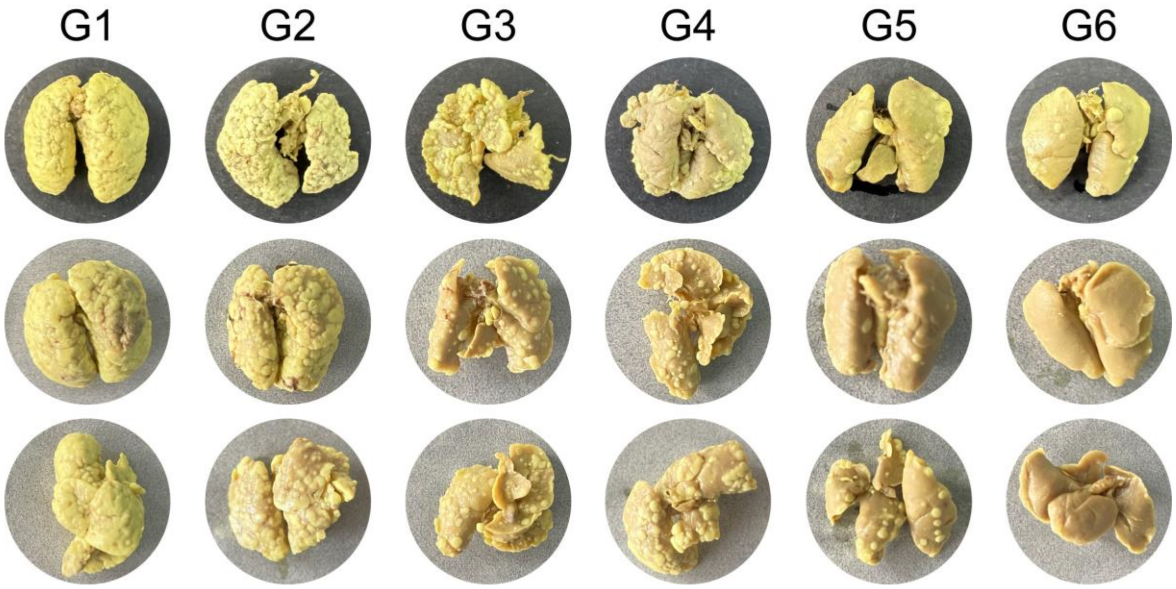

Figure S13 Images of mice lung tissue. (G1: PBS, G2: NEs+OMV, G3: OMV-Ce6+Laser, G4: NOC47-Ce6, G5: NO-Ce6+Laser, G6: NOC47-Ce6+Laser).

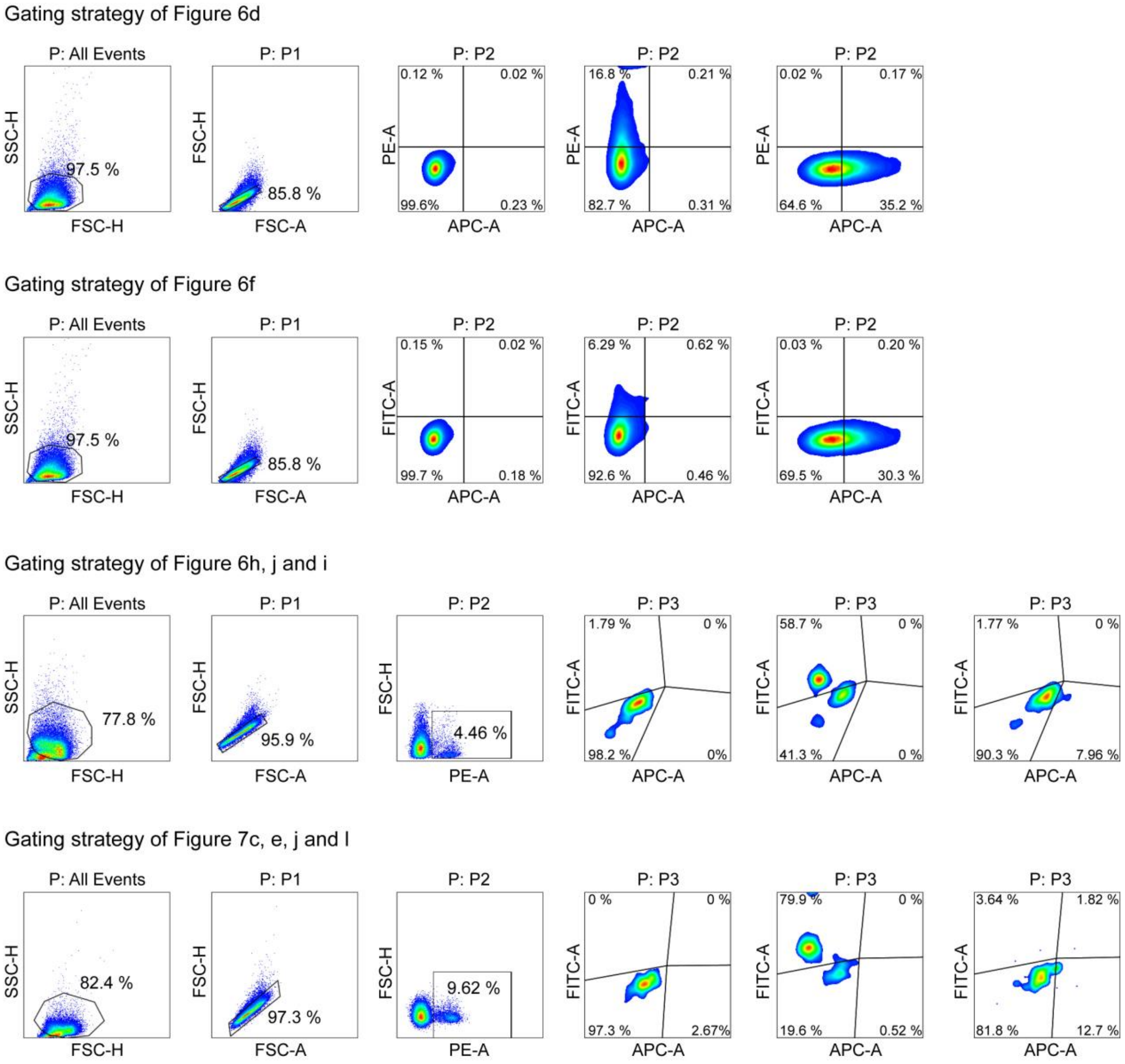

Figure S14 Gating strategy of Figure 6d, 6f, 6h, 6j, 6i, 7c, 7e, 7j and 7l.
